# Experimental study of energy‑absorbing and support characteristics of glass microsphere-filled steel tube columns under uniaxial compression

**DOI:** 10.1038/s41598-024-55562-z

**Published:** 2024-02-27

**Authors:** Jia-hui Ma, Qiu-di Sun, Shun Liu, Xiao-bin Yang

**Affiliations:** School of Emergency Management and Safety Engineering, China University of Mining and Technology-Beijing, Beijing, 100083 China

**Keywords:** Mechanical properties, Civil engineering

## Abstract

An innovative energy-absorbing and bearing structure was proposed, which incorporated the coupling of glass microspheres with a metal tube. Glass microsphere-filled steel tube (GMFST) column, consisting of external steel tube and inner glass microspheres, was expected to give full play to the energy-absorbing and load-bearing capacities of the particle while restricting particle flow from collapsing, thereby enhancing the overall structural strength. Four groups of steel tubes and the GMFST specimens were designed and subjected to axial compression tests at four different loading rates to investigate the performance of the structure. These tests aimed to analyze the deformation mode, mechanical response, and energy absorption capacity of the GMFST columns under quasi-static to low-speed compression conditions. The results indicated that the deformation process and failure mode of GMFST columns were similar to those of hollow steel tubes, albeit with a different post-buckling mode. Filling the steel tubes with glass microspheres reduced the load fluctuation range, moderated load–displacement curves, and exhibited a strain rate strengthening effect. The GMFST columns demonstrated superior energy absorption capacity, with significant increases in crush force efficiency, the averaged crush force, and the total absorbed energy, particularly in terms of subsequent support capacity. The load-increasing reinforcement properties enabled GMFST columns to overcome the limitations associated with the unstable post-buckling path of energy‑absorbing damping structure, exhibiting outstanding load-bearing performance and stability in the later stages. The results provided valuable guidelines for designing and engineering high-performance GMFST columns, serving as a new type of energy-absorbing and supporting structure.

## Introduction

Granular materials, characterized by their wide availability and stable physical properties, exhibit unique mechanical characteristics^1,2^. Granular materials have long been used as energy-absorbing or cushion device for absorbing external energy, enhancing load-bearing stability and mitigating shock waves through elastic–plastic deformation, particle collisions, friction, and fragmentation^3,4^. Granular materials exhibit complex mechanical behaviors, being both heterogeneous and capable of exhibiting characteristics of both liquids and solids^5–7^. Table 1 provides a comparison of the energy absorption mechanisms, advantages, and applications of granular materials with other common energy-absorbing materials. Constrained containers offer a solution to the challenges posed by particle flowability and excessive deformation, while simultaneously maximizing the mechanical properties and energy absorption capacity of particle materials. These containers can include hyperelastic rubber^8^, sandwich structures^9^, and tube. External constraints play a significant role in enhancing stiffness, yield strength, and energy absorption of granular materials.

**Table 1:** Comparison of commonly used energy-absorbing materials.

Energy-absorbing materials	Structure	Energy absorption mechanisms	Strengths	limitations	Applications
Tubular structure^10^	Elastic–plastic structure	Bending, tearing, buckling, etc	Featuring simplicity in structure and ease of manufacturing	Limited energy absorption capability	Widely applied
Foam materials^11^	Porous structure	Compression failure, penetration, etc	Lightweight	The physical and chemical properties may not be sufficiently stable, lower strength	Automobiles, packaging materials, sports equipment etc
Granular materials^1^	Porous structure	Friction, Elastic–plastic deformation, fragmentation, etc	Fluidity, allowing for versatile applications and shapes	Loose granular materials require external constraint	Marine engineering, noise and vibration control, underground protection projects, etc
Energy absorbing composite structures (EACS)^12^	Dispersed structure, layered structure, etc	Fiber/matrix cracking, crazing, local buckling, delamination, etc	Diverse types with excellent energy-absorbing performance	Complex fabrication and higher costs	Automotive, aircraft, protective armor, etc

To fully utilize the advantages of steel and granular material, particle-filled steel tube structures have been explored and investigated. Thin-walled metal tubes^13^ demonstrate excellent energy absorption capabilities through buckling. The shear resistance of the metal tube is limited and has a relatively weak capacity to withstand non-axial loads. The toughness and stiffness of metal materials make them suitable as external constraint materials for granular materials, effectively limiting the flow of granular materials while retaining the compressibility of granular materials.

By incorporating crushable particles within the metal tube, the particle material could jointly bear the load with the metal tube while preserving the deformation mode of the structure. Energy absorption and load-bearing stability are achieved through the deformation and fragmentation of individual particles, as well as the friction between particles. Showcasing exceptional subsequent support performance in the later stages of loading, the particles could demonstrate an increase in strength as they fracture further. This has great potential in areas that require both energy absorption and certain support capabilities, such as yielding energy-absorbing support structures in coal mines^10,14^, damping structure for soft rock support^15,16^, and underground protective engineering projects^11,17^.

Several studies have investigated the load-bearing behavior and mechanical properties of particle-filled metal tubes across a range of applications. Xu et al.^18^performed finite element (FE) numerical simulations to investigate the impact of factors such as aspect ratio on the load-bearing performance of gravel-filled steel tubes used in temporary bridge support. Yao et al.^19–21^ conducted a comprehensive investigation involving simulations and experiments to examine the mechanical properties of internally filled gravel thin-walled steel columns under different loading conditions in protective engineering projects, including axial loading, eccentric compression, and lateral impact. Jia et al.^22^ explored the potential of gravel-filled steel tubes as gob-side support bodies in coal mine tunnels and achieved promising results. Hu et al.^23^ examined the feasibility of utilizing sand-filled steel tubes as a kind of compressible support structure of reserved pier columns for gob-side entry retaining in wide roadways, specifically considering their compressive and energy-absorbing properties. In terms of energy absorption, Blanc et al.^24^ conducted a comparative analysis of the energy absorption performance of sandwich structures filled with different brittle particles subjected to explosive shock waves. Sugiyama et al.^25^ investigated the energy absorption mechanism of tube structures partially filled with particles when exposed to explosive shock waves. Lee et al.^26^ developed a theoretical formula, based on ideal elastic–plastic assumptions, for calculating the energy absorption capacity of steel tubes filled with particles. Their findings were validated through experiments and numerical simulations. Winkel et al.^27^ conducted experiments and finite element method (FEM) simulations to investigate localized flexural buckling characteristics of large-diameter soil-filled steel piles partially embedded in the seabed.

Currently, the existing research predominantly comprises experimental studies conducted in practical engineering. These studies mainly focuse on investigating the axial load-bearing behavior of particle-filled thin-walled metal tubes or studying their energy absorption mechanisms as sacrificial structures. However, there is a dearth of comprehensive research on the load-bearing performance and deformation energy absorption of particle-filled thin-walled metal tubes under axial loading. Furthermore, there is significant variation in parameters such as structural dimensions, slenderness ratios, and particle shapes, lacking in the fundamental response mechanism of granular-filled thin-walled metal tubes during the entire axial compression process.

This paper aims to explore the energy absorption stability and subsequent support capacity of composite structures comprising steel tubes filled with particles throughout the entire process of axial compression. To simplify the research parameters, small-sized steel tubes and highly circular spherical brittle glass microspheres with crushable properties were meticulously selected. Four groups of hollow steel tubes and glass microsphere-filled steel tube (GMFST) columns were subjected to axial compression tests at four different loading speeds. By comparing and analyzing the deformation mode, load–displacement curve, average force, crushing force efficiency, total energy absorption, stroke efficiency and peak load of the specimens, the mechanical response mechanism of glass microsphere-filled steel tube (GMFST) columns under quasi-static and low-speed loading is studied and the energy absorption performance is evaluated, which provides experimental data support for the popularization and application of the structure.

## Glass microsphere-filled steel tube (GMFST) columns preparation and Experimental procedures

**Figure 1:**
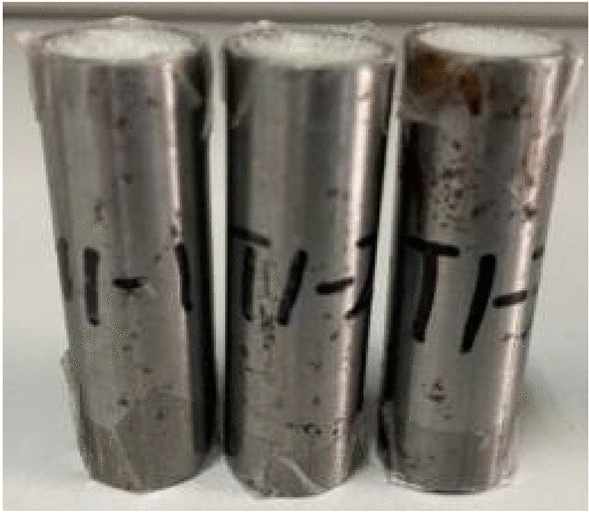
Glass microsphere-filled steel tube (GMFST) columns.

Due to limitations imposed by laboratory equipment conditions, a small-scale model was selected for this experiment. Glass microsphere-filled steel tube (GMFST) columns were composed of a thin-walled circular steel tube filled with smooth brittle glass microspheres, as illustrated in Fig. 1. Table 2 shows the relevant parameters of Q235 steel selected for the experiment. Values for Young's modulus and yield strength for Q235 steel, were obtained from widely accepted default values available in the literature and engineering standards.

**Table 2:** Parameters of the steel tube.

Symbol	Meaning	Value
E	Young’s modulus	210 GPa
σ	Yield stress	235 MPa
μ	Poisson’s ratio	0.3
H	Height	45 mm
D	Outer diameter	15 mm
T	Steel tube wall thickness	1 mm

The steel tubes used in the test were seamless steel tubes, with the steel material being Q235 carbon structural steel widely employed in the construction industry. The primary objective revolved around investigating the axial load-bearing capacity of the components, without considering the bending effect. The aspect ratio of the steel tube is defined as the ratio between the height and the outer diameter (*H*/*D*). It is important to consider the aspect ratio as excessively high values may result in the Euler buckling failure of the component rather than strength failure^28^. Conversely, the excessively low aspect ratio can affect the determination of strength due to the enhanced constraint effect of the end caps on the specimen. Therefore, a typical stub columns^29^ with an aspect ratio of three has been selected for this experiment. The diameter-to-thickness ratio of the steel tube is defined as the ratio between the outer diameter and the wall thickness (*D*/*t*). This ratio is used to measure the constraint effect of the steel tube on the core concrete in the theory of concrete-filled steel tube confinement^30^. A higher diameter-to-thickness ratio signifies a relatively thinner steel tube, resulting in a weaker constraint effect on the core concrete. Concrete-filled steel tubes with a diameter-to-thickness ratio of more than 135 or a wall thickness less than 3 mm^31^ are commonly utilized in engineering applications, referred to as thin-walled concrete-filled steel tubes. However, in this small-scale model experiment, glass microspheres necessitated more constraints to prevent particle flow and disintegration in comparison to concrete as loose particles, and excessively thinning the tube could accentuate defects, potentially impacting experimental results. A lower diameter-to-thickness ratio would lead to excessive load-bearing by the steel tube, thereby limiting the complete utilization of filled glass microspheres. Therefore, the steel tubes with a diameter-to-thickness ratio of 15 and a thickness of 1 mm were selected for this experiment. Due to their low absolute thickness, these structures can still be referred to as thin-walled steel tubes despite a high D/T ratio.

**Figure 2:**
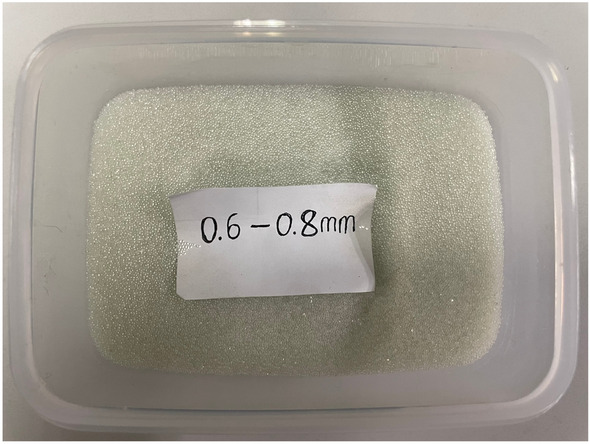
Highly spherical silica glass microspheres.

Highly spherical silica glass microspheres with a controllable and uniform size, low strength, as well as smooth surfaces, were chosen as the filling particles. This type of particle exhibited a well-established manufacturing process and widespread availability, and facilitated the repeatability of subsequent experiments. The characteristics of glass microspheres, such as lower density, a more regular shape, lower strength, and a smoother surface minimizing particle friction, make them prone to stress concentration-induced deformation and breakage. This results in a loading process characterized by a plateau stage, in contrast to sand, commonly used for filling steel tubes in temporary support for offshore engineering, where the load increases exponentially with displacement. Consequently, glass microspheres demonstrate superior energy absorption capacity and load-bearing stability, particularly under large strain conditions. Additionally, compared to foam metal or composites, due to their fluidity and stable physical and chemical properties, glass microspheres facilitate more convenient transportation and assembly. This is particularly advantageous in applications such as underground protection projects or coal mines. The density of glass microspheres was 2.6 g/cm^3^, and the initial porosity was 12.18%. The particles had a diameter ranging from 0.6 to 0.8 mm, falling within the size range of constructional medium sand, and possessed a circularity of over 90%. This selection eliminated the influence of the particles' irregular shape and the interlocking and friction effects resulting from their rough surface on stress–strain relationships and failure mechanisms of the components. Figure 2 shows the glass microspheres used in the experiment.

**Figure 3:**
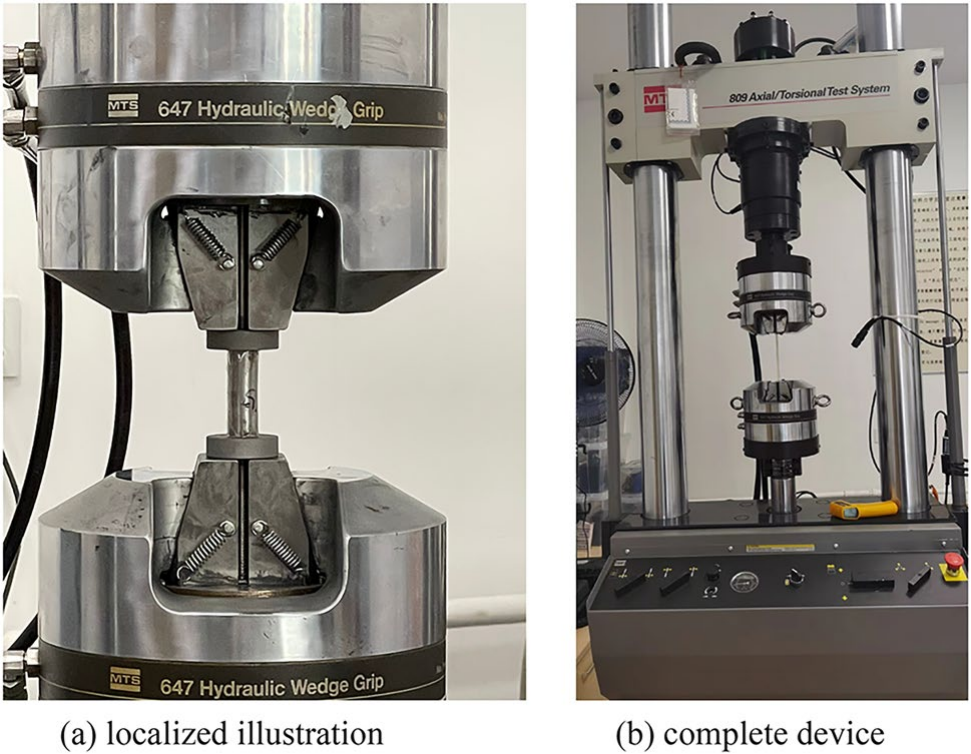
MTS hydraulic testing machine (100 kN), (**a**) localized illustration (**b**) complete device.

To minimize the experimental variability, a precision electronic scale with a 0.01 g accuracy was used to weigh 8.5 g of glass microspheres, ensuring that the weighing error for each sample is within + /− 0.03 g. The glass microsphere-filled steel tube (GMFST) columns were sealed at both ends using 0.2 mm thick PVC film. The PVC film served the purpose of confining particles to prevent spillage, enabling both the glass microspheres and the steel tube to bear the load. Other synthetic materials with good elasticity and adhesion were also suitable for this purpose. Thin-walled metal tubes and particles could be coupled without affecting the intrinsic properties of the materials using this method. The test equipment was MTS hydraulic testing machine (100 kN), as shown in Fig. 3. During the displacement-controlled loading procedure, where the load was applied in an upward direction, the force–displacement relationship data was collected by the MTS hydraulic testing machine. Simultaneously, the compression deformation process was recorded by a camera. The termination criterion for loading was set to a compression of 25 mm for the specimen or when the load surpassed the capacity of the press, ensuring a controlled and safe testing environment. The test scheme is shown in Table 3. Each group consists of two repeated samples of hollow steel tubes and three repeated samples of GMFST columns, denoted as K and T, respectively.

**Table 3:** Test scheme.

No	Numbers	Whether the specimens are filled with glass microspheres	Loading speed/(mm·s^-1^)
K0	2	No	0.0045
K1	2		0.045
K2	2		0.45
K3	2		4.5
T0	3	Yes	0.0045
T1	3		0.045
T2	3		0.45
T3	3		4.5

## Result

### Deformation mode

**Figure 4:**
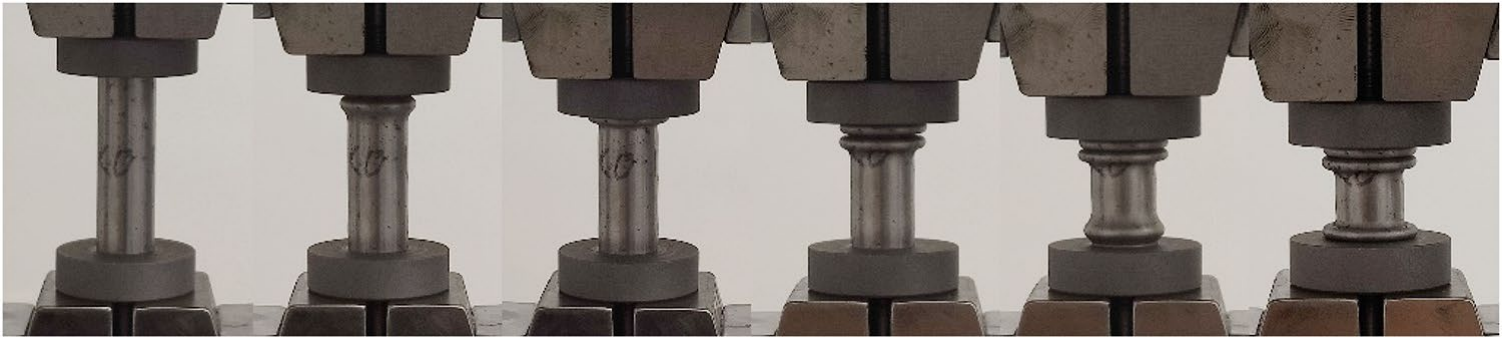
Axial compression process diagram of a hollow steel tube at a loading rate of 0.045 mm·s^-1^.

The typical deformation process of an hollow steel tube under axial pressure is shown in Fig. 4. The experimental conditions transitioned gradually from quasi-static loading to low-speed loading as the loading rate increased, while the deformation mode remained unchanged under four different loading rates. The steel tube under axial loading exhibited a typical progressive plastic buckling behavior, characterized by the formation of axially symmetric circumferential folds during both the buckling and post-buckling stages. The folding behavior exhibited distinct locality and was primarily observed at the ends of the steel tube. The steel tube underwent negligible deformation and remained in the elastic stage during the beginning of loading. Upon entering the plastic deformation stage, the fixed end began to exhibit outward bulging deformation. After the steel tube yielding, the outward bulging developed into circumferential folds, which represented the first buckling. As the loading continued, the second external bulging deformation followed below the first flexural folds and developed into a ring-shaped fold of compression, known as secondary buckling. The third buckling appeared at the loading end, with the formation and development process consistent with the first and the second buckling. The variation in buckling positions attributed to the initial defects and end effects. The deformation, yielding, and energy absorption processes of the metal tube, characterized by progressive plastic folding in an axially symmetric manner, exhibited good stability and represented an ideal failure mode.

**Figure 5:**
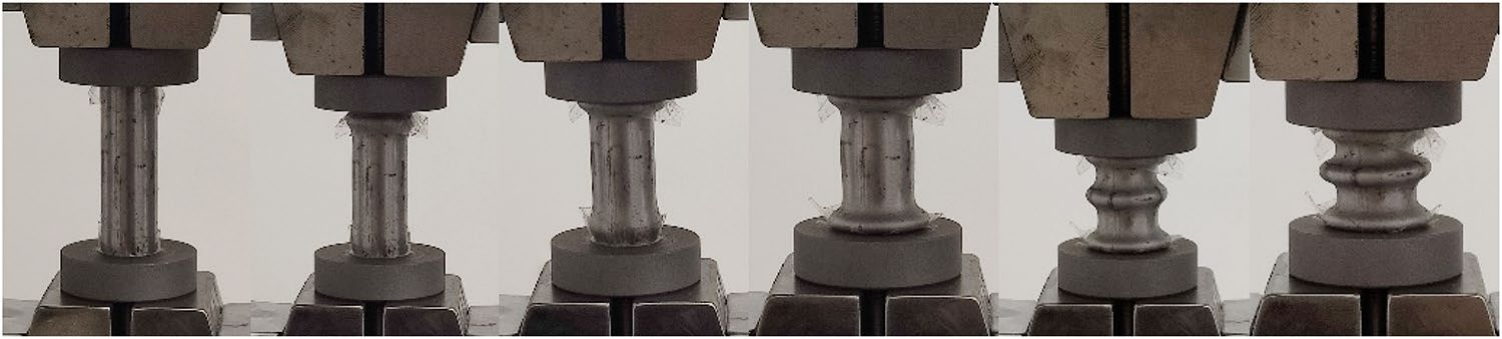
Axial compression process diagram of a glass microsphere-filled steel tube (GMFST) column at a loading rate of 0.045 mm·s^-1^.

Figure 5 illustrates the entire deformation process of the glass microsphere-filled steel tube (GMFST) column. The deformation mode of the GMFST columns followed plastic progressive buckling, but the location and sequence of circumferential folds differed from those observed in the hollow steel tube. The fixed end of the GMFST columns exhibited the first occurrence of circumferential folding. This occurrence could be attributed to the presence of a gap at the upper end of the component under the influence of gravity, where the glass microspheres not fully loaded. Another potential factor involves stress concentration at both ends of the metal tube due to a lack of reinforcement, leading to end buckling^29^. This is evidenced by a certain degree of similarity observed in the buckling locations between glass microsphere-filled steel tubes and empty steel tubes. The upper weak section of the GMFST structure yielded and deformed first, away from the loading end. The lower end of the GMFST columns exhibited the second occurrence of circumferential folding, known as the dynamic pressure head end, while the third folding appeared in the middle of the tube as the loading continued. This could have been attributed to the fact that the glass microspheres were brittle granular material, demonstrating properties of non-uniform force distribution and deformation, which were called force chains. The large deformation of particle primarily manifested as fragmentation. Contact forces and particle fragmentation were transferred from the two supporting ends of the specimen to the middle through force chains. Therefore, after the formation of the primary buckling at the upper end, the second folding occurred at the loading end, followed by tertiary flexure in the middle of the specimen.

### Load–displacement curves

**Figure 6:**
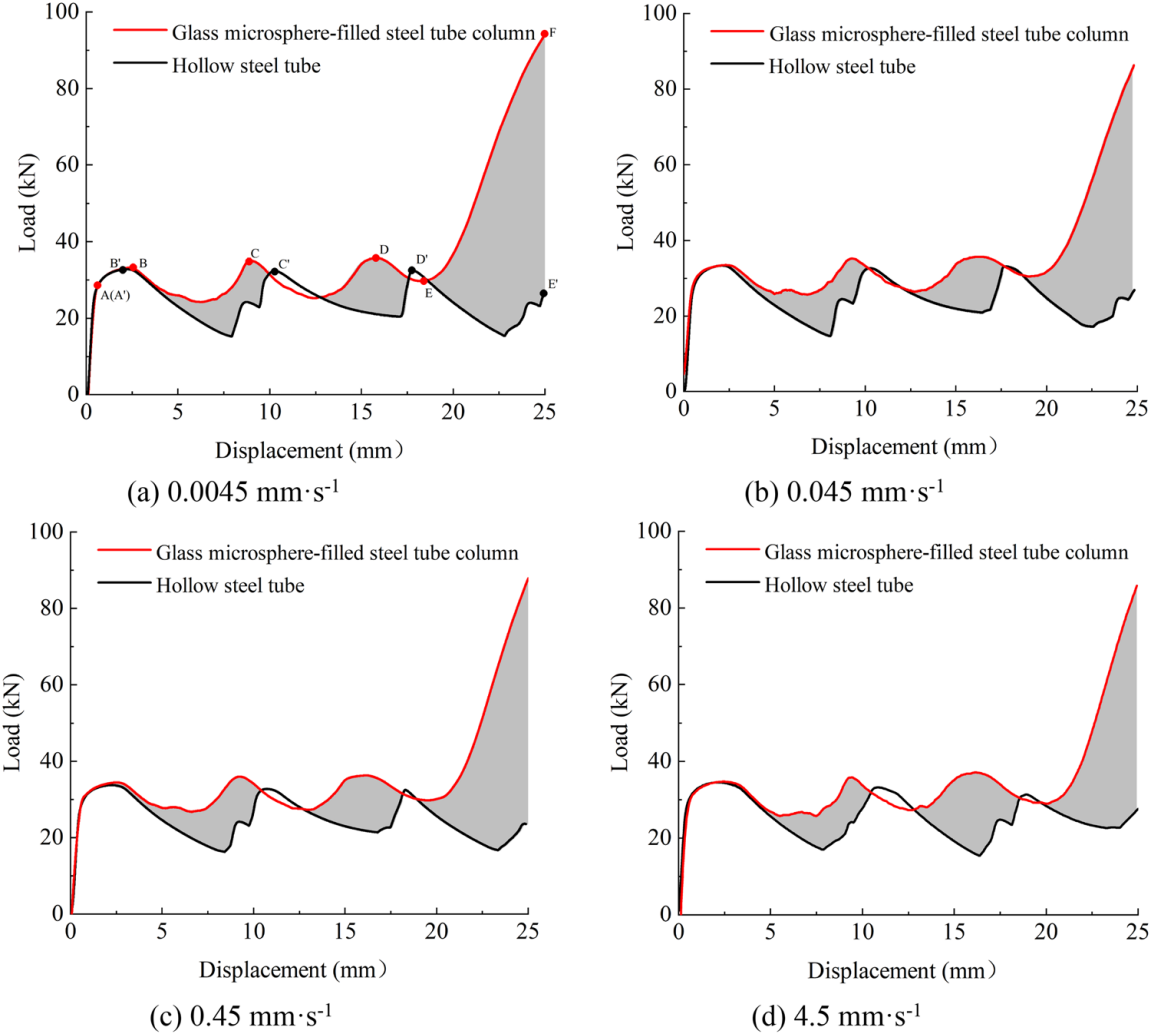
Load–displacement curves of hollow steel tubes and GMFST columns under different loading rates. (**a**) 0.0045 mm·s^-1^, (**b**) 0.045 mm·s^-1^, (**c**) 0.45 mm·s^-1^, (**d**) 4.5 mm·s^-1^.

The load–displacement curves of the hollow steel tubes and glass microsphere-filled steel tube (GMFST) columns under different loading rates are shown in Fig. 6.

The axial compression process of the hollow steel tube could be divided into three stages: the elastic stage OA', the yield strengthening stage A'B', and the buckling energy absorption stage B'C', C'D', and D'E'. The axial compression process exhibited an unstable post-buckling path, wherein the load-bearing capacity abruptly decreases after reaching the peak load. The hollow steel tube underwent energy absorption through the formation of the axisymmetric circular folding deformations, as shown in Fig. 4. The fluctuation observed in the load–displacement curve corresponded to the formation of the folds, with segments B'C', C'D', and D'E' aligning with the primary buckling, secondary buckling, and tertiary buckling shown in Fig. 4, respectively.

The filling of glass microspheres in the steel tube did not alter the correspondence between the fluctuations in the load–displacement curve and the folding formation, nor did it affect the fundamental mechanical response. The axial compression process of glass microsphere-filled steel tube (GMFST) columns remained on a stable post-yielding path, divided into four stage: elastic stage, plastic strengthening, buckling energy absorption stage, and densification stage.

1. Elastic stage OA: In the initial loading stage of the glass microsphere-filled steel tube (GMFST) columns, minimal deformation was observed. However, as the experiment progressed, the glass microspheres within the tube began to crush, resulting in an audible sound. The load–displacement curve for this stage closely overlapped the initial elastic stage OA' observed in the hollow steel tube, indicating a linear increase in load with displacement.
2. Plastic strengthening stage AB: The specimen exhibited noticeable bulging deformation, accompanied by continuous crushing of the particle. The load–displacement curve demonstrated a deceleration of growth rate. The steel tube itself started to yield, reaching the initial peak load at point B.
3. Energy absorbing stage BE: The GMFST columns demonstrated their energy absorption capacity at this stage, primarily through the buckling and folding of the outer steel tube and the deformation and crushing of glass microspheres. The load–displacement curve showed a relatively gentle slope compared with the hollow steel tube. The particle-filled steel tube underwent primary buckling at the static end, secondary buckling at the loading end, and tertiary buckling at the middle section, corresponding to the three fluctuations in the load–displacement curve (BC segment, CD segment, and DE segment). These buckling stages were accompanied by continuous glass microspheres crushing sounds.
4. Densification stage EF: The sound of glass microspheres fragmentation diminished, and the load exhibited a rapid increase. The overall stiffness of the system was noticeably heightened during this stage. Eventually, loading ceased as it exceeded the capacity of the hydraulic testing machine.

## Discussion

### influence of glass microspheres filling

Glass microspheres exhibited distinct mechanical properties different from those of a continuous metal tube. Glass microspheres relied on microscale interactions such as particle elastic–plastic deformation, collisions, friction, and particle fragmentation to dissipate energy, while the thin-walled steel tube primarily dissipated energy through plastic hinge bending and tensile dissipation during its gradual collapse.

**Figure 7:**
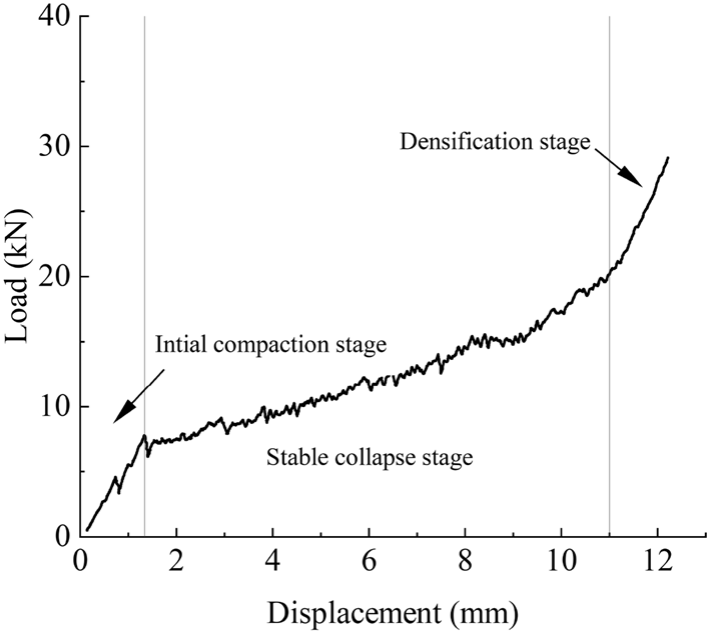
The compression force–displacement curve of glass microspheres.

The contacts between glass microspheres formed numerous load transfer pathways, typically in the form of force chains. These force chains transmitted the weight and external loads within the particle system. Figure 7 illustrates the load–displacement curves under axial loading within the context of lateral confinement compression for the selected glass microspheres in the experiment. It could be primarily divided into three stages: the compaction stage, the collapse stage, and the densification stage^32^.

Particle fragmentation altered the particle size distribution and compaction rate of the granular assembly, influencing its strength and deformation mode. The particles underwent a transient phase of elastic deformation, compressing each other and decreasing pore sizes at the beginning of loading. Due to the selected brittle and smooth glass microspheres, there was minimal friction but higher normal contact forces, resulting in stress concentration at contact points and a tendency for brittle failure as loading continued. after particle fragmentation, the voids between particles were filled with particle fragments, increasing the coordination number and compactness of the assembly. The complete fragmentation of particles led to the evolution of a certain optimal particle size distribution^33^. On the one hand, the individual particle strength experienced a sudden drop, affecting the macroscopic load-bearing capacity of the specimen during brittle particle fragmentation. On the other hand, Partial particle fragmentation resulted in particle redistribution, leading to an increased coordination number for larger particles. The enhancement of the coordination number led to forces being more evenly distributed on large particles, similar to uniform hydrostatic pressure, thereby reducing the stress concentration and deformation failure. The smaller particle fragments exhibited higher strength and load-bearing capacity. During axial compression loading, the sudden drop in strength caused by particle fragmentation coexisted with the redistribution of particle fragments and optimization of particle size distribution. These two effects counterbalanced each other, resulting in increased compactness and reduced average particle size.

Figure 6 presents a comparison of the load–displacement curves between glass microsphere-filled steel tube (GMFST) columns and the hollow steel tube at different loading rates. Filling particles substantially improved the strength of specimens. This enhancement indicated that the increase in density had a greater impact on the strength than the strength drop caused by particle fragmentation. As the loading proceeded, the particles consistently underwent fracturing, reaching the densification stage where the load-bearing capacity of the granular body rapidly increased. This phenomenon is known as strain hardening of the particle assembly, as illustrated in Fig. 7.

The filling of glass microspheres did not alter the fundamental deformation mode of the composite structure, which still exhibited a progressive plastic deformation mode. The energy absorption capacity and bearing capacity of the GMFST columns experienced significant enhancement across various stages, owing to the collaborative load-bearing and the energy absorption of the filled glass microspheres and steel tube. As loading continued, the load exhibited a fluctuating upward trend. From an energy perspective, glass microsphere-filled steel tube (GMFST) columns demonstrated not only energy dissipation through the deformation of the steel tube but also additional energy absorption owing to the friction and elastic–plastic deformation of the glass microspheres. Therefore, the GMFST columns absorbed more energy, resulting in an overall increase in the load level when they reached the same strain compared to the hollow steel tube. Additionally, the presence of the steel tube restricted the flow of the particle, while the glass microspheres provided support to the inner boundaries of the metal tube, thereby limiting the inward bending deformation. In comparison to an hollow steel tube, the plastic strengthening stage (AB) of GMFST columns was prolonged, leading to increased yield strength. The energy absorption in GMFST columns occurred through the formation of axially symmetrical folding, which corresponded to the sudden drop and subsequent gradual increase in load after the steel tube yielded, respectively. Moreover, the filling of the glass microspheres supported the load and suppressed the tendency of load drop, resulting in a smoother load–displacement curve. The support provided by the glass microspheres to the tube wall led to smaller plastic folds and an increased number of folds. Filling the steel tube with glass microspheres eliminated the secondary peak observed in the load–displacement curve of an hollow tube. The bearing capacity and stability of the structure experienced improvements. In the later stage of loading, the ductility and toughness of metal hindered particle flow and resulted in the filling of most voids between particles. Most of the inter-particle voids were filled with particle fragments, limiting the displacement of the microspheres. Consequently, the glass microsphere-filled steel tube (GMFST) columns entered the densification stage, characterized by a rapid increase in load and excellent subsequent support performance.

The inclusion of glass microspheres filling significantly improved the mechanical performance of the GMFST columns, allowing them to demonstrate superior qualities in stable energy absorption and load-bearing capabilities. Through deformation and fragmentation, glass microspheres absorbed energy in the early stage of loading, contributing to the load-bearing capability with the metal tube and optimizing the inward buckling deformation, as well as reducing the subsequent load drop during the post-buckling phase. This enhancement increased the stability of the load-bearing and energy absorption capacity of the structure. In the later stage, the strength of the component experienced substantial improvement, and the load showed an increasing-hardening trend.

### Influence of the strain rate effect

**Figure 8:**
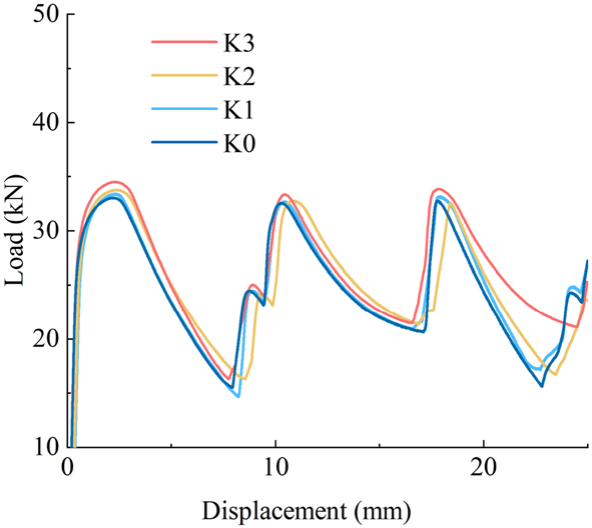
Load–displacement curves of hollow steel tubes under different loading rates.

**Figure 9:**
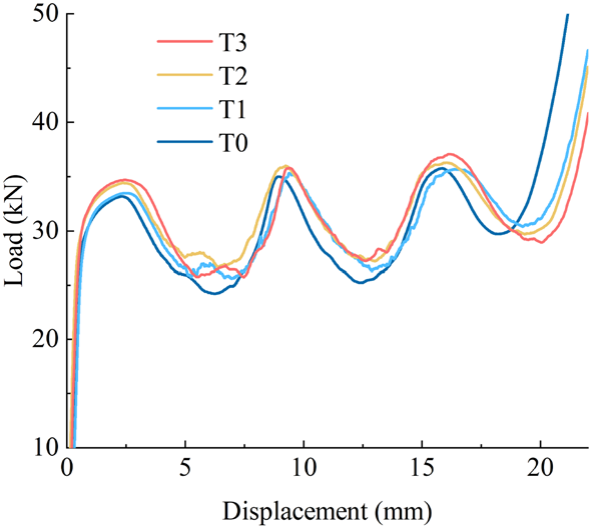
Load–displacement curves of the GMFST columns under different loading rates.

**Figure 10:**
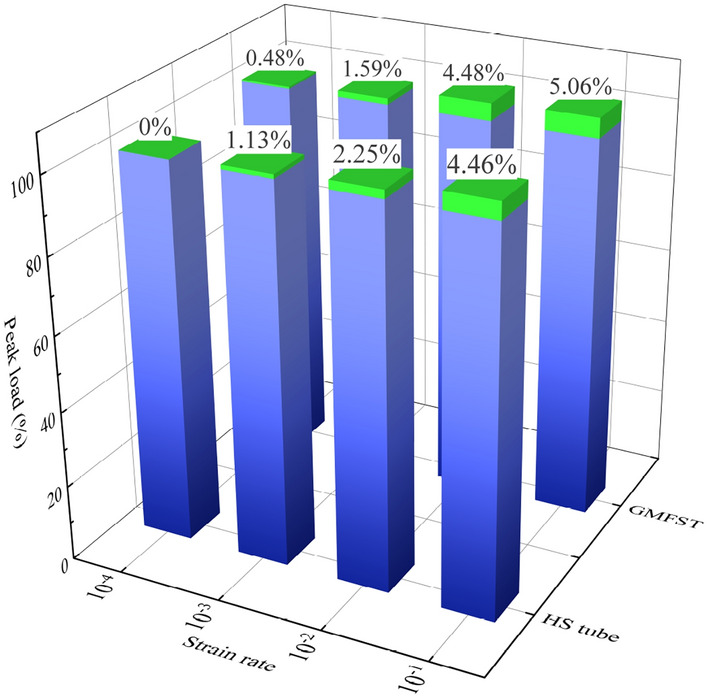
The first peak load of hollow steel (HS) tubes and the GMFST columns under different loading rates.

Figure 8 shows the load–displacement curves of the hollow steel tube from the quasi-static loading rate to the low-speed loading rate. Figure 9 shows the load–displacement curves of the glass microsphere-filled steel tube (GMFST) columns from the quasi-static loading rate to the low-speed loading rate. The strain rate effect refers to the phenomenon where the mechanical properties of the component change as the loading rate varies. From Figs. 8 and 9, it could be observed that the bearing and energy absorption capacity of both the hollow steel tube and GMFST columns exhibited a positive correlation with strain rate. Moreover, the higher the strain rate, the later the densification stage came for the GMFST columns. Figure 10 depicts the peak load growth rates under different conditions, with the growth rate of the hollow steel tube serving as the reference at a strain rate of 10^–4^. The initial peak load of both the hollow steel tube and the GMFST columns increased approximately linearly with varying loading rates. From the perspective of material properties, the macroscopic essence of the strain rate effect was the hysteresis phenomenon of deformation damage during loading. As the loading rate increased, the specimen exhibited a higher stress state at the same strain value due to delayed deformation damage^34^.

**Figure 11:**
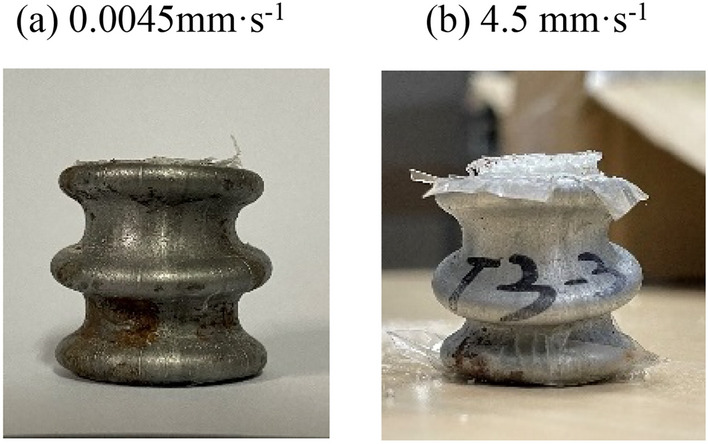
Deformation modes of the GMFST columns under different loading rates.  (**a**) 0.0045 mm·s^-1^, (**b**) 4.5 mm·s^-1^.

By observing the deformation pattern of GMFST columns after axial compression tests depicted in Fig. 11, it was noted that the strain rate increase altered the deformation mode of the glass microspheres assembly within the steel tube. After quasi-static loading, the GMFST columns formed symmetrical circumferential folds at the upper end, lower end, and middle section, with the folds developing horizontally. The large deformation of the glass microspheres primarily resulted from the closure of voids caused by particle translation^35^, indicating a progressive layer-by-layer fragmentation mode. With an increase in the loading rate, the inclined development of annular folds in the middle section of the GMFST columns indicated that the particle fragmentation inside the tube was unevenly distributed in the horizontal direction.

The strength of particles of the same kind exhibited a strong correlation with their coordination number. Due to contact with the support ends, the glass microspheres at both ends of the specimen, which experienced lower coordination numbers and asymmetric loading, underwent fragmentation initially. Subsequently, the coordination number of intact glass microspheres in the next layer was altered after the horizontal fragmentation within the first layer. As a result, a deformation and fragmentation zone, dominated by boundary effects on a macroscopic level, progressed from the two support ends towards the middle in a translational motion. The trend of the occurrence and development of the particle crushing position corresponded with the position and order of the axisymmetric annular folds of the GMFST columns. Particle slippage and rearrangement dominated the macroscopic deformation of the specimen as the loading rate increased^32,36^. The glass microspheres rapidly compacted, and their fragmentation mode was influenced by initial structural defects and uneven local grading at high strain rates. Randomly distributed concentrated fracture zones appeared in the middle region, resulting in uneven fragmentation within the same horizontal plane. Nevertheless, the progressive layer-by-layer fragmentation dominated by boundary effects persisted. Therefore, the glass microsphere-filled steel tube (GMFST) columns exhibited axisymmetric annular collapse at both ends and oblique collapse in the middle under high strain rate conditions.

### Energy absorption and support capacity of the glass microsphere-filled steel tube (GMFST) columns

The mechanical response of the entire axial bearing process of glass microsphere-filled steel tube (GMFST) columns was investigated by measuring the energy absorption and subsequent support capacity during the post-buckling stage. The energy absorption performance of the structure was evaluated by studying the average crushing force, crush force efficiency, total absorbed energy and stroke efficiency of GMFST columns^37^. A comparison with the hollow steel tube was conducted to examine the changing trend of peak load corresponding to multi-level buckling of the GMFST columns under different strain rates. The macroscopic impact of the micro-scale glass microsphere filler crushing process on the mechanical properties of the component was analyzed, providing data support for practical engineering applications.

#### Average crushing force and crush force efficiency

The average load, denoted as $\overline{P}$, was defined as the average value of the load during the BE stage, which corresponded to the post-stress hardening and pre-densification phases shown in Fig. 6a. $\overline{P}$ was expressed by Eq. (1):

1 $$\overline{P} = \frac{{\mathop \smallint \nolimits_{0}^{{S_{e} }} P(x){\text{d}}x}}{{S_{e} }}$$

where $S_{e}$ is the effective densification displacement, representing the total displacement of the structure from the the initial peak load to the initiation of the densification stage, $P(x)$ is the instantaneous load at the displacement of $x$.

The crushing force efficiency, referred to as $CFE$, is defined as the ratio between the initial peak load $P_{\max }$ and the average load $\overline{P}$. In an ideal energy-absorbing structure, the crushing force efficiency should be 1. $CFE$ was caculated using the Eq. (2):

2 $$CFE = \frac{{P_{\max } }}{{\overline{P} }}$$

**Figure 12:**
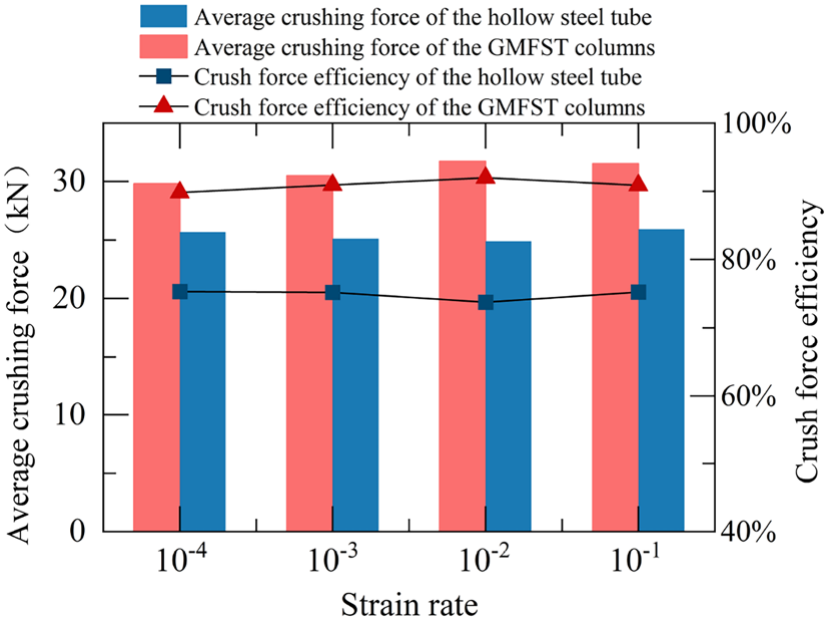
Average load and crush force efficiency of hollow steel tubes and the GMFST columns under different loading rates.

The average load and crushing force efficiency of the GMFST columns and the hollow steel tubes at different strain rates are shown in Fig. 12. After filling the tube with glass microspheres, both the average load and crushing force efficiency of the GMFST columns significantly improved. The GMFST columns exhibited a 16%, 21%, 27%, and 21% increase in average load compared to the hollow steel tube at the same strain rates. The average crushing force efficiency of the hollow steel tube was 74.8%, which increased to 90.9% after filling it with glass microspheres, indicating a significant enhancement in energy absorption stability. The average load and crush force efficiency primarily reflected the stable energy absorption capacity of the specimen during compression. The filling of glass microspheres greatly optimized the energy absorption stability of the structure, while the strain rate had little effect on the energy absorption stability of the structure.

#### Total absorbed energy

The total absorbed energy of the specimen is defined as the total area under the load–displacement curve of the GMFST columns before densification, caculated using the Eq. (3):

3 $$E = \mathop \smallint \nolimits_{o}^{\delta } P(x){\text{d}}x,$$

where $\delta$ is the total displacement before the dencification stage.

To facilitate comparison, the densification displacement of the GMFST columns was selected as the termination point of the energy absorption stage of the hollow steel tubes when calculating the energy absorption performance parameters, as the hollow steel tube did not reach the densification stage.

**Figure 13:**
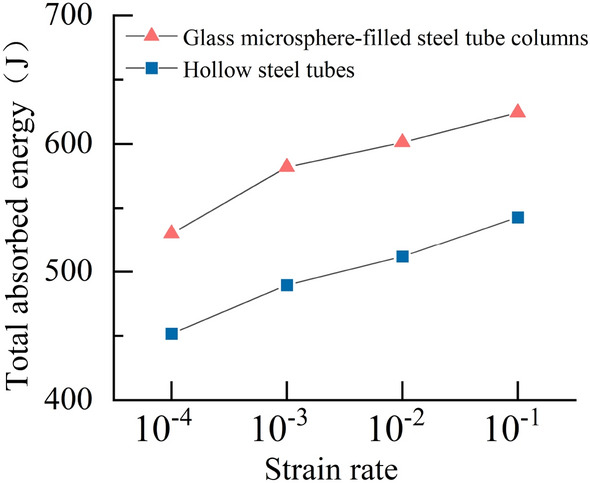
Total absorbed energy of hollow steel tubes and the GMFST columns under different loading rates.

The comparison of total absorbed energy between the specimens is shown in Fig. 13. After filling with glass microspheres, the energy absorption of the specimens significantly increased. Compared to the hollow steel tube at the same loading rate, the GMFST columns exhibited an increase in energy absorption of 78.29 J, 91.98 J, 88.93 J, and 81.58 J, with an average improvement of 17.12%. The energy absorption of the structure mainly included the deformation energy of the steel tube, the deformation energy of the glass microspheres, the energy from glass microspheres fragmentation, and the energy dissipation. The energy absorption capacity of the specimens was also influenced by the strain rate. With the increase of strain rate from 10^–4^ to 10^–1^, the energy absorption capacity of the hollow steel tube and the GMFST columns increased by 91.04 J and 94.33 J respectively.

#### Stroke efficiency

The stroke efficiency, referred to as $SE$, is a dimensionless quantity that measures the utilization of a material as an energy-absorbing structure^36^. It is defined as the Eq. (4).

4 $$SE = \frac{\delta }{L},$$

**Figure 14:**
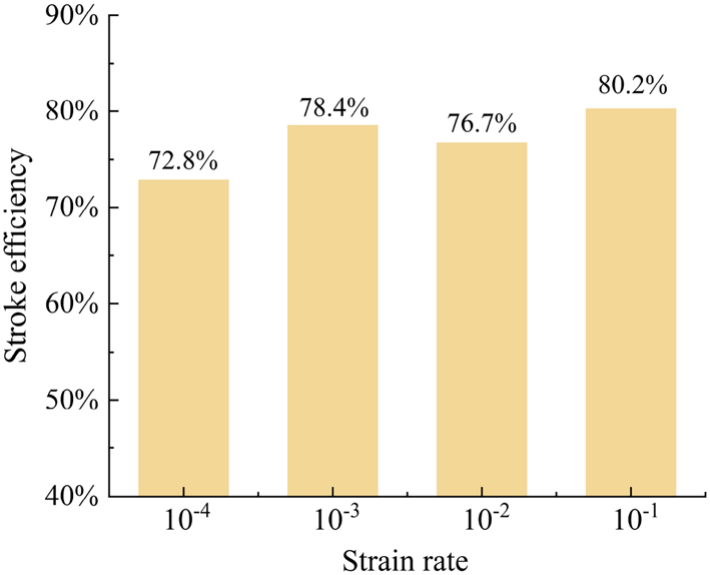
Stroke efficiency of the GMFST columns under different loading rates.

A higher stroke efficiency indicates a better utilization of the structure as an energy-absorbing system, with a later densification stage. Figure 14 shows the comparison of stroke efficiency for the GMFST columns at different strain rates. The stroke efficiency of the GMFST columns displayed an upward trend, surging from 72.8 to 80.2% with escalating loading rates, underscoring the manifestation of strain rate effects. The elevation in loading velocity curtailed the energy transmission efficiency of the force chain among the glass microspheres within the GMFST columns, leading to heightened energy dissipation. Since primarily energy dissipation was through the deformation and fragmentation of the glass microspheres, higher loading rates required greater external force input to achieve the same level of deformation and fragmentation, resulting in delayed densification so that improved stroke efficiency.

#### Subsequent support capacity

**Figure 15:**
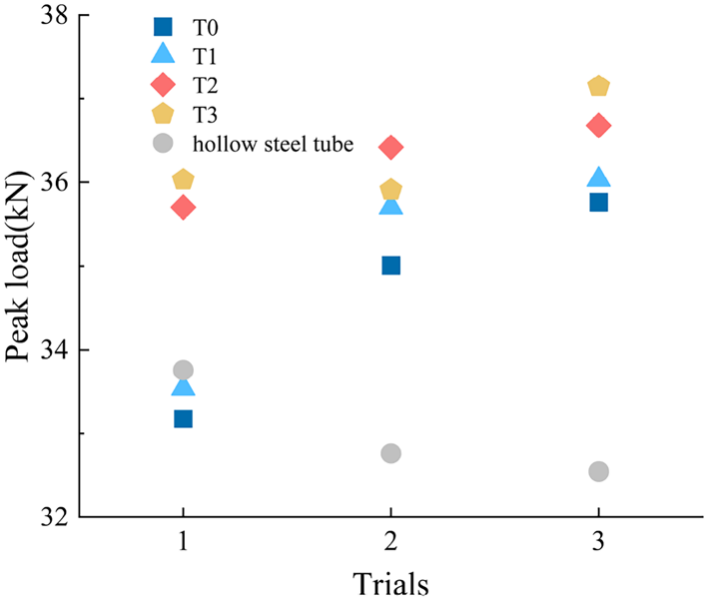
The changes of peak load of the GMFST columns under different loading rates.

In contrast to other energy-absorbing damping structures that typically encountered strength reduction after yielding, the strength of the glass microsphere-filled steel tube (GMFST) columns progressively heightened throughout the loading process, showcasing its exceptional subsequent support performance. Figure 15 provides a representation of the peak load variation before the densification stage for GMFST columns at different strain rates, along with a comparison to hollow steel tubes.

During the post-buckling stage of the hollow steel tube, referred to as the formation of the first collapse, the load progressively rose to the peak load, which was lower than the initial yield strength of the hollow steel tube. Moreover, the peak load diminished with the continuous formation of the annular shrinkage. This decrease occurred because the hollow steel tube could only withstand the load through the non-plastically deformed portion, and the folding introduced axisymmetric geometric defects, resulting in an overall reduction in strength. Conversely, the peak load corresponding to each folding stage in GMFST columns exhibited a continuous upward trend during the loading process. At different strain rates, the peak load increased by 2.58 kN, 2.48 kN, 2.18 kN, 2.45 kN compared to the initial peak load of the GMFST columns. This was because the glass microspheres continuously fragmented during loading, leading to a smaller average radius and a higher degree of densification before further significant deformation and fragmentation occurred. Smaller particle sizes corresponded to higher strength, and the smaller particles distributed the load more evenly within the steel tube, thereby increasing the strength of the component and counteracting the strength reduction caused by the plastic yield of the external steel tube. From a macroscopic perspective, this was manifested as a constant increase in strength and an ascending trend in the peak load as the loading continued. Upon reaching a point of adequate particle fragmentation, in comparison to the state before loading, there was a notable reduction in the average particle size. Simultaneously, the particle size distribution became consistent, signaling the establishment of a stable coordination number. Subsequent deformation and fragmentation encountered increasing challenges, highlighting the initiation of the densification phase, during which the bearing capacity of the glass microsphere-filled steel tube (GMFST) columns significantly augmentation.

The distinctive mechanical characteristic of subsequent support provided the GMFST columns with coupling advantages of energy absorption capability through post-buckling deformation and excellent load-bearing support ability, making them a promising combination of the traditional rigid support structure and energy-absorbing damping structures and opening up a broad spectrum of potential applications.

## Conclusion

Twelve glass microsphere-filled steel tube (GMFST) columns and eight hollow steel tube columns were subjected to monotonic axial compression tests to investigate their energy absorption and load-bearing capabilities, as well as the mechanical response under axial compression. The primary parameters considered in the study were the strain rate and whether the glass microspheres were filled. Based on the experimental results, the following conclusions can be drawn.

1. The deformation mode of the GMFST columns was similar to that of the metal tube, exhibiting progressive plastic buckling deformation and forming axisymmetric ring-shaped folding.
2. Both the GMFST columns and empty steel tubes exhibited a pronounced strain rate strengthening effect, essentially a hysteresis phenomenon of damage deformation during loading. The failure mode of the GMFST columns changed with the increasing loading rate.
3. Filling the structure with glass microspheres effectively enhanced its energy absorption capacity. The load–displacement curve of the GMFST columns became smoother, the inward collapse of the tube was mitigated, the particles were constrained.The introduction of glass microspheres led to a significant increase in the crushing force efficiency, average crushing load, and total absorbed energy of the structure.
4. The GMFST columns exhibited an load-increasing reinforcement properties. Once the glass microspheres were fully crushed and densified, the strength of the specimens significantly improved, demonstrating their unique ability for subsequent support. The GMFST columns possessed coupling advantages of energy absorption through post-buckling deformation and excellent load-bearing support ability, making them a promising combination of traditional rigid support structures and energy-absorbing damping structures.

## Data Availability

The datasets generated during and/or analysed during the current study are available from the corresponding author on reasonable request.
